# A BAC-based integrated linkage map of the silkworm *Bombyx mori*

**DOI:** 10.1186/gb-2008-9-1-r21

**Published:** 2008-01-28

**Authors:** Kimiko Yamamoto, Junko Nohata, Keiko Kadono-Okuda, Junko Narukawa, Motoe Sasanuma, Shun-ichi Sasanuma, Hiroshi Minami, Michihiko Shimomura, Yoshitaka Suetsugu, Yutaka Banno, Kazutoyo Osoegawa, Pieter J de Jong, Marian R Goldsmith, Kazuei Mita

**Affiliations:** 1 Insect Genome Research Unit, National Institute of Agrobiological Sciences, Owashi, Tsukuba, Ibaraki 305-8634, Japan; 2 Genome Project Department, Tsukuba Bank, Mitsubishi Space Software Co., Ltd, Takezono, Tsukuba, Ibaraki 305-8602, Japan; 3 Laboratory of Insect Genetic Resources, Faculty of Agriculture, Kushu University, Fukuoka 812-8581, Japan; 4 Children's Hospital Oakland Research Institute, 52nd Street, Oakland, California 94609, USA; 5 Biological Sciences Department, University of Rhode Island, Kingston, Rhode Island 02881-0816, USA

## Abstract

**Background:**

In 2004, draft sequences of the model lepidopteran *Bombyx mori *were reported using whole-genome shotgun sequencing. Because of relatively shallow genome coverage, the silkworm genome remains fragmented, hampering annotation and comparative genome studies. For a more complete genome analysis, we developed extended scaffolds combining physical maps with improved genetic maps.

**Results:**

We mapped 1,755 single nucleotide polymorphism (SNP) markers from bacterial artificial chromosome (BAC) end sequences onto 28 linkage groups using a recombining male backcross population, yielding an average inter-SNP distance of 0.81 cM (about 270 kilobases). We constructed 6,221 contigs by fingerprinting clones from three BAC libraries digested with different restriction enzymes, and assigned a total of 724 single copy genes to them by BLAST (basic local alignment search tool) search of the BAC end sequences and high-density BAC filter hybridization using expressed sequence tags as probes. We assigned 964 additional expressed sequence tags to linkage groups by restriction fragment length polymorphism analysis of a nonrecombining female backcross population. Altogether, 361.1 megabases of BAC contigs and singletons were integrated with a map containing 1,688 independent genes. A test of synteny using Oxford grid analysis with more than 500 silkworm genes revealed six versus 20 silkworm linkage groups containing eight or more orthologs of *Apis *versus *Tribolium*, respectively.

**Conclusion:**

The integrated map contains approximately 10% of predicted silkworm genes and has an estimated 76% genome coverage by BACs. This provides a new resource for improved assembly of whole-genome shotgun data, gene annotation and positional cloning, and will serve as a platform for comparative genomics and gene discovery in Lepidoptera and other insects.

## Abstract 2

An integrated map of the Bombyx mori genome has been constructed using 361.1 Mb of BAC contigs and singletons together with a genetic map containing 1689 independent genes and synteny among Apis, Tribolium, and Bombyx was examined.

## Background

Genome analysis of insects has moved quickly in recent years, in part because insects are so widespread and diverse, and elucidating their characteristic biological phenomena will yield enormous resources for basic science, agriculture, and industry. Complete genome sequences have been published for 12 *Drosophila *spp. [1-3], *Anopheles gambiae *[4] and *Apis mellifera *[5], and that of *Tribolium castaneum *will appear shortly [6]. In 2004, the draft sequence of *Bombyx mori *was reported independently by groups in Japan [7] and China [8]. Because of relatively shallow genome coverage (3× and 6×, respectively) using the whole-genome shotgun (WGS) sequencing method, the silkworm genome is still somewhat fragmented, with an average contig length of under 6 kilobases (kb). This makes it difficult to identify and annotate genes effectively, or to obtain a global view of the silkworm's general and unique features as a model for Lepidoptera.

Well developed genetic resources for silkworm include more than 400 described morphologic and biochemical mutants [9], affecting such characters as chorion (eggshell) composition and structure; embryo development; larval cuticle transparency, pigmentation, segment identity, and body shape; hemolymph proteins; cocoon color, shape, and texture; and adult fertility, egg laying behavior, eye color, and wing pattern. These have been assigned to more than 200 loci on linkage maps. Additionally, molecular linkage maps composed of various markers including about 1,000 random amplified polymorphic DNAs [10,11], about 250 RFLPs (restriction fragment length polymorphism) [12-14], 545 amplified fragment length polymorphisms [15], more than 500 simple sequence repeats [16,17], more than 500 single nucleotide polymorphism (SNPs) [18], and more than 400 sequence tagged sites for cloned genes and expressed sequence tags (ESTs) [19] have been constructed. Three bacterial artificial chromosome (BAC) libraries [20,21] and more than 185,00 ESTs [22-24] are also available. For a more complete genome assembly and analysis, and to take full advantage of these extensive resources, it is of great importance to combine genetic maps with physical map information. This can be accomplished by connecting genetic mapping data to BAC clones; this is a well established approach that has not been employed in silkworm on a genome-wide basis.

Our aim in the present study was to conduct a complete genome analysis. We report here an integrated map between a high-density SNP genetic map and a physical map of BAC contigs using the following strategy: extension of the previous SNP linkage map using BAC end sequences to produce a second-generation map containing 1,755 SNP markers; construction of BAC contigs using two methods, namely restriction digest fingerprinting of BAC clones and hybridization of ESTs to a BAC library on high-density replica (HDR) filters; and assignment of 1,082 ESTs to existing linkage groups by Southern analysis using a nonrecombining female informative backcross. Finally, we searched for orthologs among 1,688 genes on the updated silkworm linkage maps and tested the level of synteny with honey bee and beetle chromosomes using the Oxford grid method.

## Results

### Linkage map construction

We previously constructed a linkage map by surveying the segregation patterns of 534 SNPs detected in 190 first-generation backcross (BC_1_) individuals from a single pair mating between a p50T female and an F_1 _male (p50T female × C108T male) [18]. Based on the analysis of additional data with Mapmaker/exp (version 3.0 [25]; LOD [log of the odds] score 3.0) using the same mapping panel, we successfully positioned a total of 1,755 SNPs on an expanded linkage map. The SNP markers segregated into 28 linkage groups, with a total recombination length of 1,413 cM. We assigned 26 of the SNP linkage groups to classical silkworm chromosomes 1 to 26, defined by morphologic markers (for example, cocoon color and larval markings) and protein polymorphisms (for instance, hemolymph proteins), as reported previously [18]. Unambiguous morphologic markers are not yet available for the remaining two classical linkage groups; therefore, we anchored linkage group 27 to a reference gene (vitellogenin), and arbitrarily defined linkage group 28.

**Figure 1:**
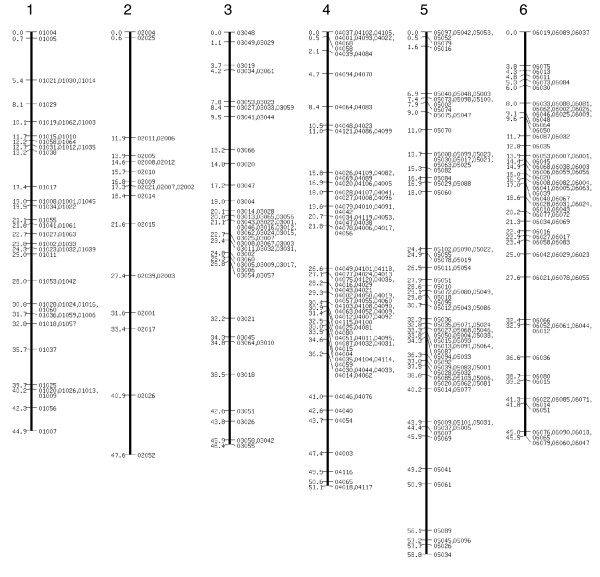
SNP linkage map comprising 1,755 markers: linkage groups 1 to 6. For additional details see Silkworm Genome Research Program [26]. SNP, single nucleotide polymorphism.

**Figure 2:**
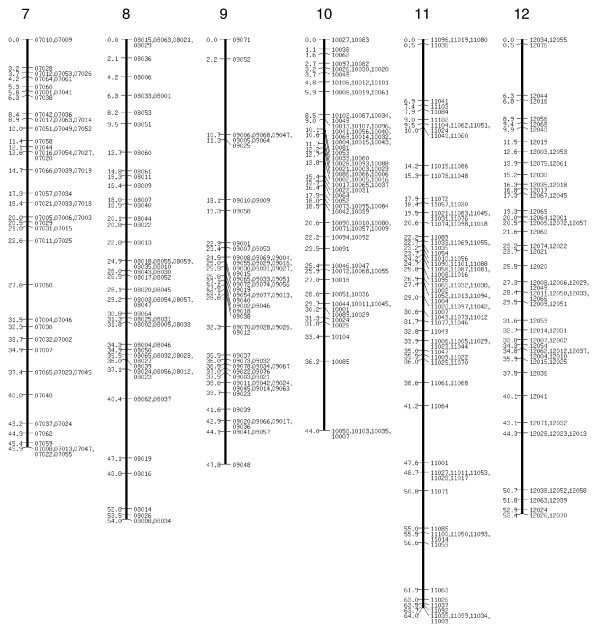
SNP linkage map continued: linkage groups 7 to 12. SNP, single nucleotide polymorphism.

**Figure 3:**
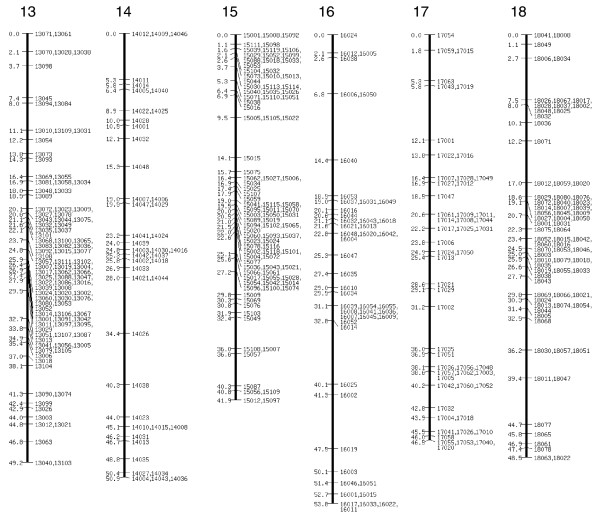
SNP linkage map continued: linkage groups 13 to 18. SNP, single nucleotide polymorphism.

**Figure 4:**
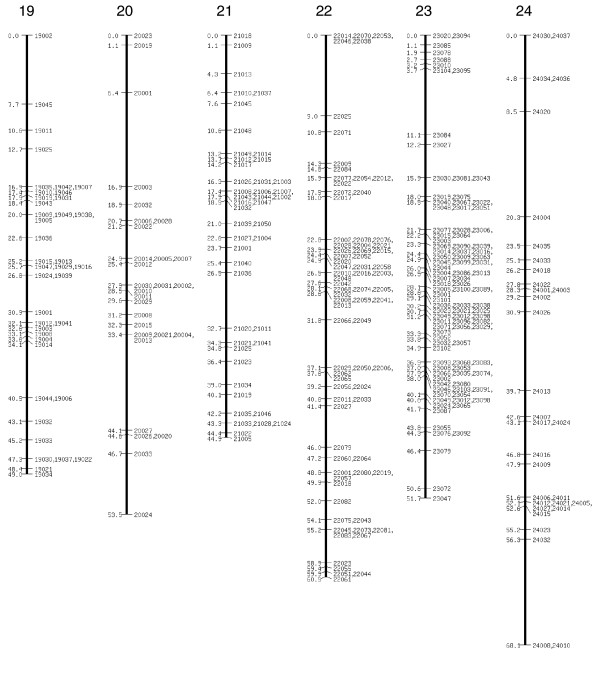
SNP linkage map continued: linkage groups 19 to 24. SNP, single nucleotide polymorphism.

**Figure 5:**
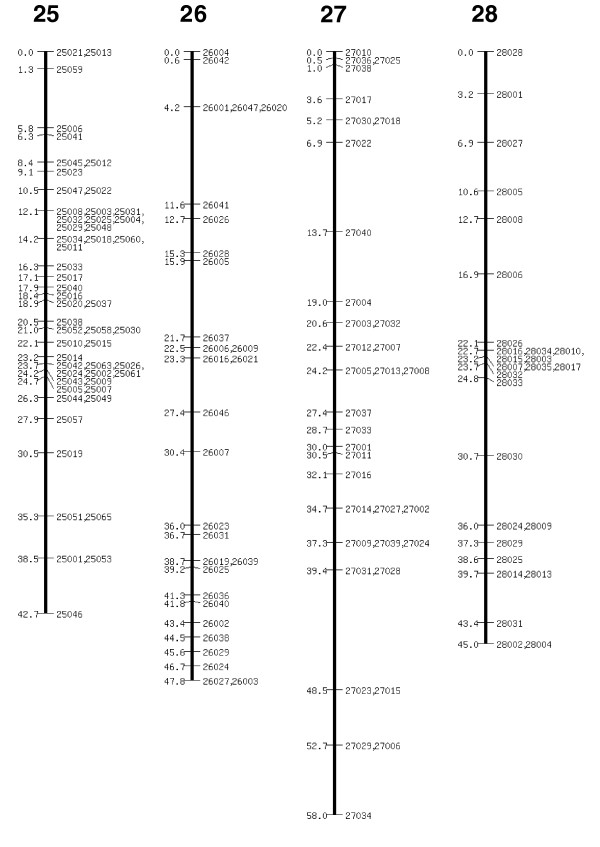
SNP linkage map continued: linkage groups 25 to 28. SNP, single nucleotide polymorphism.

The extended SNP linkage map is illustrated in Figures 1 to 5[26] (Additional data file 1 for details of each marker, including BAC accession number). Basic map parameters are significantly improved in this version. The number of markers per linkage group varies from 20 (group 2) to 105 (group 4; Figure 1), and the recombination length for each linkage group ranges from 42.2 cM (group 15; Figure 3) to 68 cM (group 24; Figure 4). In the previously reported SNP map [18], the minimum and maximum number of markers were seven (groups 26 and A; Figure 5) and 32 (group 10; Figure 2), and the linkage map lengths ranged from 27 cM (group 20; Figure 4) to 64 cM (group 11; Figure 2). The number of markers for individual linkage groups in the revised map increased proportionally to the threefold rise in the total number of markers, but the extension of the linkage maps remained relatively small, because of a higher marker density. The average distance between the markers is 0.81 cM, which is much improved compared with that of the previous map (2.5 cM). The markers are not evenly distributed throughout the linkage map, and so different regions are more densely or sparsely populated. The number of gaps with lengths exceeding 10 cM decreased to five from 14 in the previous map, and the largest gap length decreased to 12.4 cM from 21.3 cM.

### BAC contig construction by DNA fingerprinting

We fingerprinted a total of 81,024 BACs from three BAC libraries, each made with a different restriction enzyme (Table 1), using the large-scale agarose gel-based restriction fingerprinting method [27,28]. We used the computer program FPC V6.0 [29,30] to assemble BAC contigs from the BAC fingerprints. We performed preliminary tests to determine appropriate tolerance and cut-off value parameters, and adopted values of 3 and 1 × e^-12^, respectively. A lower stringency condition produced larger contigs, but it also increased the risk for false contigs because of a high density of repetitive sequences.

**Table 1:** Characteristics of BAC libraries used in this study

BAC library	Vector	Cloning site	Number of clones	Mean insert size (kb)	Clone coverage^a^
*Eco*RI-BAC	pBACe3.6	*Eco*RI	36,864	168	13×
*Bam*HI-BAC	pBeloBAC11	*Bam*HI	21,120	165	7.3×
*Hind*III-BAC	pBAC-lac	*Hind*III	23,040	125	6.1×

All libraries were constructed with strain p50T using mixed sexes. ^a^To estimate the genome coverage of each library, we used a genome size of 476 megabases. BAC, bacterial artificial chromosome.

Of the clones fingerprinted, we deleted 2,246 BACs during fingerprint editing because of insert-empty clones, having too many bands because of possible contamination, or too few bands (fewer than five). In addition, we removed 1,152 BACs from contigs assembled by the FPC program because they formed an extensive false contig, in which several constituting BACs were assigned to different chromosomes by independent BAC-fluorescence *in situ *hybridization experiments (data not shown). The false BAC contig is likely to have formed from transposon-rich regions with similar restriction fingerprints, which were difficult to remove even by the use of high-stringency assembly conditions (namely, with a cut-off below 1 × e^-12^).

The resulting physical map contained 6,221 contigs, among which 782 major contigs included eight or more BAC clones, as summarized in Table 2 (Additional data file 2). The total length of the 782 major contigs was 376 megabases (Mb), which corresponds to 79% of the silkworm genome (476 Mb measured by flow cytometry; Johnston JS, personal communication).

**Table 2:** Summary of fingerprinted BAC contigs

Number of fingerprinted BAC clones (FPC)	Number of singletons	Number of contigs	Number of major contigs with eight or more BACs	Total length major BAC contigs (Mb)
78,778	47,274	6,221	782	376

BAC, bacterial artificial chromosome; Mb, megabases.

We evaluated the reliability of the predicted BAC contigs using two approaches. First, using BAC end sequences [31] we designed primer sets for the BAC clones belonging to a putative contig to determine the presence or absence of the sequence in each BAC clone independently by PCR. Using this method, we found mis-assemblies in six contigs out of the 782 major BAC contigs; we could not determine whether nine additional contigs, which we denote as 'doubtful', were correctly formed. In most cases of doubtful contigs, only one BAC clone bridged two well defined clusters of overlapped BACs. Such a BAC clone is likely to be chimeric. Therefore, we removed such chimeric and doubtful BAC clones from the 15 BAC contigs involved, which no longer represented a contradiction in our evaluation.

The second approach was a comparison with SNP markers. If a BAC contig included more than two SNPs, then those markers should be positioned around the same locus. A total of 128 BAC contigs contained more than two SNP markers, among which we found contradictions in 11 BAC contigs. We checked the 11 contigs by PCR using primers designed from BAC end sequences, as described above. However, we were unable to resolve the contradictions because most of the BAC end sequences in question corresponded to repetitive transposon sequences, and PCR provided similar bands even if BACs in the false contig belonged to different chromosomes. This situation reinforced the supposition that BAC clones forming all or most of the false contigs were derived from transposon-rich domains that produced similar restriction band patterns.

### Integration of the linkage map and BAC contigs

Altogether we mapped 581 BAC contigs containing 6,061 BACs onto 28 chromosomes through BAC clones containing SNP markers common to the linkage map. The length of mapped singletons and BAC contigs calculated for each chromosome is shown in Table 3. A total of 361.1 Mb are covered by BACs, which corresponds to 76% coverage of the genome.

**Table 3:** Summary of integrated SNP linkage maps and fingerprinted BAC contigs

Linkage group	Number of Markers	Recombination length (cM)	Number of mapped contigs	Sum of contig lengths (Mb)	Number of BACs in contigs	Total length BAC singletons and contigs (Mb)
1	53	44.9	8	2.2	32	9.5
2	20	47.4	8	2.1	60	4.1
3	57	46.4	24	8.3	304	12.8
4	105	50.6	32	11.2	431	20.8
5	94	58.6	31	9.2	258	18.1
6	84	45.5	20	6.5	246	15.4
7	62	45.9	20	5.9	177	12.7
8	60	53.8	15	4.6	131	11.6
9	68	47.8	22	6.6	211	14.0
10	91	43.8	32	10.0	302	18.4
11	98	63.9	27	8.5	259	19.9
12	68	53.3	33	11.6	405	16.7
13	104	49.0	31	10.5	325	21.9
14	44	50.8	19	6.2	224	9.7
15	99	42.2	32	10.3	359	20.1
16	49	53.9	19	5.8	182	10.4
17	54	46.5	22	6.8	208	11.4
18	77	48.0	20	6.7	273	15.5
19	40	49.2	15	4.6	144	8.4
20	29	53.5	13	4.4	192	6.6
21	46	44.9	17	5.2	163	9.7
22	77	60.2	24	8.6	309	16.5
23	99	51.7	33	9.7	290	20.0
24	32	68.0	10	2.8	81	6.4
25	54	42.8	20	5.8	141	11.1
26	29	47.6	10	2.9	104	5.7
27	35	58.2	14	4.8	158	8.3
28	27	45.0	10	2.9	92	5.4

Total	1,755	1,413.4	581	184.7	6,061	361.1

BAC, bacterial artificial chromosome; Mb, megabases; SNP, single nucleotide polymorphism.

### Mapping of EST markers onto SNP markers and BAC contigs

Screening of mapped BACs harboring functional genes by HDR filter hybridization using EST probes is a powerful tool for positional cloning. A large EST database is available in silkworm [22-24]. Therefore, in addition to using DNA fingerprinting for the construction of BAC contigs, we employed the 'overlapping' method with EST markers, whereby BAC clones arrayed on HDR filters are subjected to large-scale screening by hybridization with individual, nonredundant ESTs to identify clones carrying single copy sequences. This approach helped confirm BAC contigs and allowed us to identify functional genes on the combined physical-genetic map. For screening we used the RPCI-96 BAC library, consisting of 36,864 clones with an average insert size of 168 kb, which corresponds to 13× redundancy.

From the number of positive BAC clones screened by HDR filter hybridization, we could roughly estimate whether the probe cDNA was a single-copy or multiple-copy gene, or contained a repetitive sequence. Table 4 summarizes the results of the EST hybridization experiments. For EST mapping, we employed only putative single-copy genes, based on filter hybridization characteristics and the number of positive hits per filter. For a single-copy gene, approximately 13 BACs should give hybridization signals. Of 692 putative single-copy genes identified by this procedure, we were able to assign 523 EST markers to chromosomes by identifying BACs common to fingerprinted contigs that had been integrated with the genetic map via SNP markers (Additional data file 1). We identified an additional 353 ESTs on the mapped contigs by BLAST search of BAC end sequences. Of these 152 were duplicates, yielding a total of 724 mapped single-copy genes. For confirmation of the initial map assignments, we designed specific primer sets to amplify expected ESTs on HDR filter-screened BACs by PCR (Additional data file 1).

**Table 4:** Summary of BAC HDR filter hybridizations with EST probes

Number of probes detected	Number of single copy genes	Number of 2-copy genes	> 3 copy genes	Repetitive sequences
1,960	692 (35.3%)	585 (29.8%)	211 (10.8%)	469 (23.9%)

We used two high-density replica (HDR) filters containing a total of 36,864 independent bacterial artificial chromosome (BAC) clones for hybridization experiments (13× genome coverage). We did not include the expressed sequence tag (EST) if one of the two filters gave poor results because of smearing or high background, or if the two filters gave imbalanced numbers of positive signals (for instance, the ratio of positive signals between two filters exceeded 5). Cut-off values used to define gene copy number were as follows: single copy gene, 2 to 15 positive signals; two copy gene, 16 to 29 positive signals; > 3 copy gene, > 30 positive signals; and repetitive sequence, > 50 copies. We mapped 523 single copy genes represented by these hybridized ESTs via single nucleotide polymorphism (SNP) markers.

### Linkage analysis of ESTs using RFLPs

In parallel with the HDR filter hybridization experiments, we carried out RFLP analysis of segregants from a backcross between an F_1 _female (p50T × C108T) and a C108T male using a common set of ESTs as hybridization probes. The lack of meiotic crossing over in females results in complete linkage, enabling fast and efficient chromosome assignment of large numbers of markers using small segregant populations [13]. We assigned a total of 1,082 ESTs to linkage groups by RFLP analysis (Table 5 and Additional data file 3); of these, 118 ESTs were mapped in duplicate using HDR filter hybridization or BAC end SNPs. In addition to providing independent confirmation of linkage assignments on the integrated SNP-physical map, the linkage assignment of 964 new ESTs will enable future annotation and evaluation of mapped scaffolds in the WGS assembly now in progress [7,8] (Mita K, Xia Q, personal communication).

**Table 5:** Summary of ESTs assigned to linkage groups

Linkage group	Number of ESTs mapped by filter hybridization	Number of ESTs mapped by BAC end sequences	Number of ESTs mapped by RFLPs	Total number of independent ESTs^a^
1	17	7	21	36
2	15	8	25	37
3	16	10	26	43
4	25	18	37	73
5	37	18	50	87
6	16	8	46	65
7	12	20	34	57
8	16	16	50	71
9	19	13	45	65
10	29	15	47	77
11	38	26	54	95
12	22	9	42	64
13	18	16	42	67
14	6	6	20	30
15	46	32	65	113
16	24	11	30	54
17	17	10	56	75
18	15	14	39	62
19	12	8	40	59
20	13	6	38	50
21	12	12	36	52
22	21	14	59	86
23	27	19	48	78
24	10	7	31	39
25	19	13	34	55
26	8	9	15	28
27	11	7	32	44
28	2	5	20	26

Total	523	357	1,082	1,688

^a^Duplicates were removed from the total number of independent expressed sequence tags (ESTs). BAC, bacterial artificial chromosome; RFLP, restriction fragment length polymorphism.

### Synteny with other insects

Altogether we assigned 1,688 independent silkworm genes to 28 linkage groups (Table 5). Of these, there is positional information for 724 genes, whereas 964 ESTs are simply assigned to a chromosome. We tested these 1,688 genes for orthology between silkworm and other model holometabolous insects for which complete genome data were available, notably *A. mellifera, T. castaneum, A. gambiae*, and *Drosophila melanogaster*, in order to compare the syntenic relationships among them. We did not conduct an analysis of synteny relative to dipteran chromosomes because of their small number, which would produce many false connections, and their high rate of chromosome rearrangement [32-34], which would probably reduce the number of significant syntenic blocks within chromosome arms or segments. Among 1,688 silkworm genes, we found 769 orthologs for *A. mellifera *and 790 for *T. castaneum *that could be used for a test of synteny. We then checked the distribution of silkworm gene orthologs in honey bee and beetle chromosomes (Additional data file 3).

**Figure 6:**
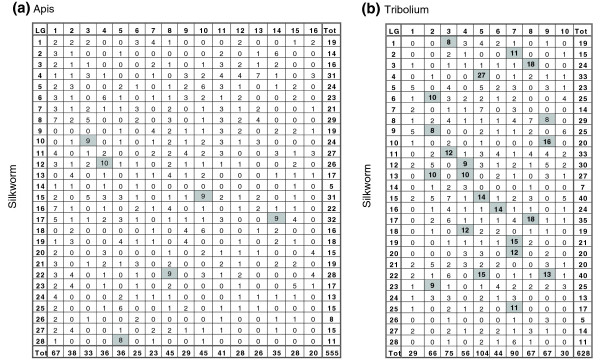
Oxford grids. Shown are Oxford grids displaying a matrix of cells comparing the number of orthologous genes on chromosomes of two species. **(a) **Silkworm-honey bee comparison. **(b) **Silkworm-*Tribolium *comparison. Shadowed cells show high synteny conservation (eight or more orthologs).

Figure 6 presents Oxford grids showing the number of shared orthologs mapped in honeybee (555 total) and beetle (628 total) on silkworm chromosomes. Based on the simplifying assumption that chromosomes are of equal length and shared orthologs are uniformly distributed throughout the genome, the ratios of observed versus expected silkworm orthologs per chromosome should be 1.0. The ratios of shared orthologs per chromosome fell in the range of 0.75 to 1.3 in the 16 honey bee chromosomes [35] and 0.8 to 1.2 in the ten beetle chromosomes [36], which is consistent with the possibility that the mapped silkworm genes were randomly sampled for both genomes.

A striking feature of this analysis is that a very poor syntenic relationship exists between silkworm and honey bee (Figure 6a) compared with that of silkworm and beetle (Figure 6b). Thus, only six chromosomes of the silkworm genome, namely linkage groups (LGs) 10, 12, 15, 17, 22 and 28 (shaded in Figure 6a), share high syntenic conservation with honey bee chromosomes (eight or more shared orthologs). In contrast, 20 silkworm chromosomes, namely LGs 1, 2, 3, 4, 6, 8, 9, 10, 11, 12, 13, 15, 16, 17, 18, 19, 20, 22, 23 and 25 (shaded in Figure 6b), exhibit high correspondence of synteny conservation relative to beetle chromosomes. Consistent with these findings, honey bee chromosomes had fewer shared silkworm orthologs than did beetle (average 9 versus 12.7 orthologs per chromosome), and the fraction of shared orthologs in syntenic groups was much smaller (0.07 in honey bee versus 0.35 in beetle). For most of the orthologous genes, homology scores between silkworm and honey bee were similar to those between silkworm and beetle, indicating that the rate of evolutionary change was comparable, whereas those for Diptera were frequently lower (data not shown).

## Discussion

The 3.3-fold increase in SNP markers from 534 in our previous study to 1,755 in the present one represents a significant improvement in the quality of the linkage map. This is reflected by a roughly proportional decrease in average marker spacing from 2.5 cM to 0.81 cM, or approximately 270 kb. Despite the increase in markers, the total recombination length only increased from 1,305 cM [18] to 1,413.4 cM, or 1.08-fold, suggesting that map lengths determined by our method are reaching asymptotes. This may reflect factors that would reduce detection of crossing over in a relatively small mapping population (190 individuals), such as a high frequency of double crossovers and the presence of gene-dense regions along chromosomes. The recombination map length obtained in this study is approximately half that obtained in projects using random amplified polymorphic DNA (3,229 cM [19]) and simple sequence repeat (3,432 cM [16]) markers, which is not easily explained and awaits integration of markers from independent studies into a single linkage map. The total length of the genome covered by mapped BACs amounted to 361.1 Mb, corresponding to 76% genome coverage in the present integrated map, in which BACs and BAC contigs produced by fingerprinting were aligned on the SNP linkage map through common BAC markers (Table 3). This suggests that the present SNP markers cover most of the silkworm genome.

Two linkage groups, 11 (64 cM) and 24 (68 cM), are significantly longer than the others. One explanation for this is the high frequency of double crossovers in these two chromosomes. The double-crossover frequency of group 24 is especially high, at 7.9% (15/190) of all detected recombinants, with group 11 next, at 6.3% (12/190). By contrast, double crossover frequencies of the other linkage groups are in the range of 0% to 2%. Consistent with these observations is that these two chromosomes are observed cytologically to be longer than the others [37]. In addition, group 11 contains the nucleolus organizer region, and group 24 has strongly stained DAPI (4,6 diamidino-2-phenylindole, dihydrochloride) regions, which are speculated to be heterochromatin [37]. Such special chromosome structures might cause the observed high frequency of crossing over, yielding longer recombination lengths for the two linkage groups. Further investigation is required to clarify the role and mechanism of double crossovers in the two longest linkage groups, as well as to determine the strain and population distribution of recombination frequency variation. In potentially related observations, polygenic factors have been proposed to be responsible for differences in recombination frequency between morphological markers on linkage group 2 in artificially selected high and low recombination silkworm lines [38,39].

The difference in relative divergence times between honey bee, beetle, and silkworm based on phylogenomic analyses [40,41] provides a partial explanation for the difference in synteny that we observed, with longer evolutionary time enabling more chromosome rearrangements. Another potential contributing factor is the average genome-wide recombination rate, which is 2.97 cM/Mb (1,413 cM/476 Mb) for silkworm in our study. This is within the same order of magnitude reported for *T. castaneum *(2.87 cM/Mb), *D. melanogaster *(1.59 cM/Mb), and *A. gambiae *(0.84 cM/Mb), whereas a much higher rate, 19 cM/Mb, was reported for *A. mellifera *[42]. This, together with changes in chromosome number, could account for more extensive gene reorganization in honey bee than beetle relative to silkworm.

Of interest is that we found a larger number of silkworm orthologs on conserved linkage groups in both honey bee and beetle than an earlier study, which was based on the 6× WGS assembly (six and four orthologs listed in synteny in the *Tribolium *and *Apis *genomes, respectively [41]). A likely explanation is that the genome-wide scaffold reported here is much more extensive than the initial WGS genome assembly, presenting a larger chromosome-anchored dataset for comparison. Recent studies reveal extensive synteny among lepidopteran insects [19,43,44]. The extent of microsynteny, or conservation of close linkage among shared orthologs within chromosomes, remains an important question for understanding patterns of genome evolution among Lepidoptera and other holometaboous insects, which will await a more complete assembly and annotation of the silkworm genome.

## Conclusion

The integrated genetic-physical map with 76% genome coverage by BACs provides a powerful basis for construction of minimum BAC tiling paths, which is an essential resource for positional cloning. The average distance between SNP markers of 270 kb, in combination with new sequencing data generated for a forthcoming re-assembly of the initial WGS data [7,8], will allow us to obtain super-scaffolds of megabase order in the near future. Our assignment of nearly 10% of predicted silkworm genes [7,8] to 28 chromosomes will not only facilitate construction of accurate scaffolds and annotation of the silkworm genome, but also provide a valuable resource for testing microsynteny and gene discovery in Lepidoptera and other insects.

## Materials and methods

### Silkworm strains and crosses

The inbred silkworm strains p50T and C108T, maintained at the University of Tokyo, were used as parental strains for the mapping panels. For linkage map construction, the same population of 190 segregants of a single-pair backcross (BC_1_) between a p50T female and an F_1 _male (p50T female × C108T male) was used as the first-generation SNP map [18].

### Genomic DNA extraction

Genomic DNA of parental strains, F_1 _individuals and segregants from the female-informative backcross was isolated from whole bodies of fifth instar larvae after removing midguts and hemolymph, as described in a previous report [14]. Genomic DNA of individual BC_1 _segregants was isolated from whole pupae using DNAzol (Invitrogen Japan K.K., Tokyo, Jpn) after freezing in liquid nitrogen and homogenization with stainless steel beads.

### Survey of the SNPs between p50T and C108T

For the linkage map construction, SNPs, including small base insertions and deletions, were used. The SNPs were identified in a large number of PCR amplicons that were synthesized using primers designed from the data obtained by BAC end sequencing, as reported previously [18]. Briefly, for each end sequence, we designed a PCR primer pair using Primer3 [45], and performed PCR amplification of the genomic DNA of the parental (p50T and C108T) and F_1 _strains with ExTaq (TaKaRa Bio Inc., Otsu, Shiga, Jpn), using the manufacturer's instructions. We detected the presence of SNPs in these amplicons by sequencing all three genotypes and analyzing the resulting traces using PolyPhred [46].

### Linkage map construction

Linkage map construction was carried out as previously reported [18]. Genomic DNA of the same 190 BC_1 _segregants was amplified with primer sets corresponding to the SNPs detected between parent strains, and polymorphism in the segregants was determined (Additional data file 1). Segregation patterns were analyzed using Mapmaker/exp (version 3.0 [25]) with the Kosambi mapping function [47]. For genotyping polymorphisms, we directly sequenced PCR amplicons from BAC end regions and used a fluorescent polarization dye terminator SNP detection assay [18,48] in parallel.

### BAC libraries

Three BAC libraries prepared using different restriction enzymes were emplyed in the present study; their characteristics are summarized in Table 1. A BAC library was constructed from genomic DNA of strain p50T fifth instar day 3 posterior silk glands partially digested with *Eco*RI [21], designated RPCI-96 (RP96), and is available from BACPAC Resources of the Children's Hospital Oakland Research Institute [49]. The *Hind*III-BAC library was prepared using *Hind*III partial digestion of the same p50T strain DNA [20]. The *Bam*HI library, similarly prepared with *Bam*HI using DNA from a strain derived from the same line as p50T (Wu C, Goldsmith M, personal communication) was purchased from the Laboratory for Plant Genomics and GENEfinder Genomic Resource of Texas A&M University [50]. All three libraries were fingerprinted for the construction of genome-wide BAC contigs. The RPCI-96 and *Bam*HI-BAC libraries were used for SNP analysis; the *Eco*RI-BAC library was used for EST mapping by HDR filter hybridization.

### BAC HDR filter hybridization with EST probes

RPCI-96-BAC clones arrayed in duplicate in a specific pattern onto nylon membranes (HDR filters) were obtained from BACPAC Resources of the Children's Hospital Oakland Research Institute [49]. Labeling, hybridization, and detection were performed using the ECL Direct Nucleic Acid Labeling and Detection System (GE Heathcare UK Ltd., Little Chalfont, Buckinghamshire, UK), in exact accordance with the manufacturer's instructions [21]. Probes derived from ESTs were obtained by PCR amplification of plasmid cDNA inserts using universal sequencing primers corresponding to the plasmid vectors (see below). EST library construction and analysis have been reported [22].

### BLAST search

The BLASTN [51] search of 1,755 BAC end sequences was carried out against an in-house collection of *B. mori *cDNA/ESTs containing 185,765 sequences. In this search, the e value (a probability cut-off value) was set to 1 × e^-50 ^and no complexity filter was used. The cDNA/ESTs of *B. mori *were retrieved from NCBI-GenBank Flat Files (release 159.0) with a custom Perl script (Additional data file 1).

### Fingerprinting analysis and BAC contig construction

DNA fingerprinting analysis was used to construct BAC contigs. The detailed protocol is reported in Marra and coworkers [27], and we followed the steps described by Osoegawa and coworkers [28] exactly. BAC DNA was isolated from 1.2 ml cultures of LB medium containing chloramphenicol using an automated method (PI-1100; Kurabo Industries Ltd, Osaka, Japan) and dissolved in 50 μl of TRIS-EDTA buffer. Approximately 6 ng (3 μl) of DNA was digested in a 20 μl reaction containing five units of *Eco*RI at 37°C overnight. When the digestion was completed, 4 μl of 6× loading buffer (15% Ficoll [Sigma-Aldrich Japan K. K., Tokyo, Jpn], 0.25% bromophenol blue, and 0.25% xylene cyanol) was added and 3 μl of *Eco*RI-digested DNA was separated on a 1% agarose gel in 1ξ Tris-Acetate-EDTA buffer at 90 V for 15 minutes, then at 45 V for 12 hours. Electrophoresis was carried out in Horizon 20-25 electrophoresis tanks (Life Technologies, Gaithersburg, MD, USA) at 16°C. Size markers containing 12.5 ng/μl Analytical Marker DNA, Wide Range Ladder (Promega K. K., Tokyo, Jpn), and 2 ng/μl Marker V (Roche Diagnostics K.K., Tokyo, Jpn) were loaded in every fifth lane. After electrophoresis, the gel was stained in 500 ml of 1:1,000 dilution of SYBR Green 1 (FMC BioProducts, Rockland, ME, USA) in 1× TAE for 30 minutes and scanned using a Molecular Imager FX (Bio-Rad Laboratories Inc., Tokyo, Jpn). The gel image was analyzed using Image 3.9d software [52,53], followed by FPC v.6.0 software [29,30].

### RFLP linkage analysis

Southern blotting was used to assign EST clones to linkage groups. After sequencing and homology search [22], unique clones were amplified by PCR with plasmid-specific primers to encompass the inserts such as M13 reverse or T3/M13M3, M13M4, or T7 primer sets. Amplified EST DNA was labeled using the ECL Direct Nucleic Acid Labeling and Detection System (Amersham-Pharmacia Biotech). BC_1 _segregants from a single female informative pair mating ([p50T × C108T] female × C108T male) were used for analysis of linkage, using 16 samples per probe. When the DNA of a sample was used up a new sample was taken from the same family and assayed with a reference set of anchor loci to determine the inheritance pattern for each linkage group [13]. To test polymorphism, digestion was carried out on 2.4 μg parental DNA with one of six restriction enzymes, *Bam*HI, *Bgl*II, *Eco*RI, *Hin*dIII, *Kpn*I, or *Sac*I, and subjected to 0.8 % agarose gel electrophoresis for 16 hours at 14°C. Gel treatment for denaturation, depurination, neutralization, blotting onto nylon membranes (nylon membranes, positively charged; Roche Diagnostics K. K., Tokyo, Jpn), and probe labeling and hybridization were performed in exact accordance with the manufacturer's instructions. Selection of suitable enzymes to detect the RFLPs between the two parents and the analysis of linkage in the BC_1 _segregants was carried out using the method of Kadono-Okuda and coworkers [13].

## Abbreviations

BAC = bacterial artificial chromosome; BC_1 _= first-generation backcross; BLAST = basic local alignment search tool; EST = expressed sequence tag; HDR = high-density replica; kb = kilobases; LG = linkage group; Mb = megabases; PCR = polymerase chain reaction; RFLP = restriction fragment length polymorphism; SNP = single nucleotide polymorphism; WGS = whole-genome shotgun.

## Authors' contributions

KY designed and performed SNP analysis using BAC end sequences and construction of the SNP linkage map. JNo performed fingerprinting of whole BAC clones and construction of BAC contigs by FPC. KK designed RFLP linkage analysis and performed synteny analysis by Oxford grid and EST hybridization experiments. JNa participated in SNP analysis. MS participated in RFLP analysis and EST hybridization experiments. SS performed all DNA sequencing. HM contributed in construction of the integrated map. MS participated in integrated map construction and Oxford grid analysis. YB participated in the assignment of the SNP linkage groups to the classical linkage groups with visual markers. KO contributed in BAC library construction, characterization of BACs, and BAC contig construction with FPC. PJ also contributed in BAC library construction and provided the knowledge on BAC contig construction with FPC. MG provided the overall knowledge of linkage maps and participated in manuscript preparation. KM conducted the whole project, and participated in EST hybridization experiments and manuscript preparation. All authors read and approved the final manuscript.

## Additional data files

The following additional data are available with the online version of this paper. Additional data file 1 is a table listing 1,755 SNP markers. Additional data file 2 is a table listing 6,221 BAC contigs constructed by fingerprinting 81,024 BACs from three BAC libraries made with different restriction enzymes. Additional data file 3 is a table listing 1,688 genes mapped onto 28 silkworm linkage groups by EST hybridization, RFLP, and BAC end sequence analyses, and their orthologs in *Apis *and *Tribolium*.

## Supplementary Material

Additional data file 1 Columns 1 and 2 list the chromosome number and positional information of each SNP marker, respectively. Columns 4 and 5 list PCR primer sequences designed from BAC ends (column 7) for SNP analysis. Columns 10 and higher list ESTs identified in end sequences of BAC markers by homology search. Click here for file

Additional data file 2 Column 1 lists the contig identification number (ID). Columns 2 and 3 list the contig size and number of BAC clones assigned to a contig, respectively. The remaining columns list the BAC clones composing each contig. Click here for file

Additional data file 3 Columns 1 and 2 list the *Bombyx *cDNA clone name and accession number, respectively. Column 3 lists the LG on which the gene is located. Columns 4 and 5 list the accession number of the *Apis *ortholog and its LG, respectively, and columns 6 and 7 list the accession number and LG for corresponding orthologs of *Tribolium*. Columns 8, 9, and 10 list the procedure used for mapping each gene in *Bombyx*. Click here for file
